# The distinct features of microbial ‘dysbiosis’ of Crohn’s disease do not occur to the same extent in their unaffected, genetically-linked kindred

**DOI:** 10.1371/journal.pone.0172605

**Published:** 2017-02-21

**Authors:** Umer Zeeshan Ijaz, Christopher Quince, Laura Hanske, Nick Loman, Szymon T. Calus, Martin Bertz, Christine A. Edwards, Daniel R. Gaya, Richard Hansen, Paraic McGrogan, Richard K. Russell, Konstantinos Gerasimidis

**Affiliations:** 1 School of Engineering, University of Glasgow, Glasgow, United Kingdom; 2 Warwick Medical School, University of Warwick, Warwick, United Kingdom; 3 Human Nutrition, School of Medicine, Dentistry and Nursing, College of Medical, Veterinary and Life Sciences, University of Glasgow, Glasgow Royal Infirmary, Glasgow, United Kingdom; 4 Institute of Microbiology and Infection, University of Birmingham, Birmingham, United Kingdom; 5 Gastroenterology Unit, Glasgow Royal Infirmary, Glasgow, United Kingdom; 6 Department of Paediatric Gastroenterology, Hepatology and Nutrition, Royal Hospital for Children, Glasgow, United Kingdom; "INSERM", FRANCE

## Abstract

**Background/Aims:**

Studying the gut microbiota in unaffected relatives of people with Crohn’s disease (CD) may advance our understanding of the role of bacteria in disease aetiology.

**Methods:**

Faecal microbiota composition (16S rRNA gene sequencing), genetic functional capacity (shotgun metagenomics) and faecal short chain fatty acids (SCFA) were compared in unaffected adult relatives of CD children (CDR, n = 17) and adult healthy controls, unrelated to CD patients (HUC, n = 14). The microbiota characteristics of 19 CD children were used as a benchmark of CD ‘dysbiosis’.

**Results:**

The CDR microbiota was less diverse (p = 0.044) than that of the HUC group. Local contribution of β-diversity analysis showed no difference in community structure between the CDR and HUC groups. Twenty one of 1,243 (1.8%) operational taxonomic units discriminated CDR from HUC. The metagenomic functional capacity (p = 0.207) and SCFA concentration or pattern were similar between CDR and HUC (p>0.05 for all SCFA). None of the KEGG metabolic pathways were different between these two groups. Both of these groups (HUC and CDR) had a higher microbiota α-diversity (CDR, p = 0.026 and HUC, p<0.001) with a community structure (β-diversity) distinct from that of children with CD.

**Conclusions:**

While some alterations were observed, a distinct microbial ‘dysbiosis’, characteristic of CD patients, was not observed in their unaffected, genetically linked kindred.

## Introduction

Crohn’s disease (CD) clusters within families, yet more than 150 host genes collectively explain only a minor fraction of the overall disease risk[1]. A discordant risk of CD in monozygotic twins[2], points to the importance of non-genetic factors, including diet and the intestinal microbiota. A distinct gut microbiota in CD has consistently been described in the literature[3], and while the evidence is in favour of a primary role in the aetiology of the disease, no specific bacterial taxon has yet been convincingly implicated as fundamental to CD pathophysiology. It is also unclear whether microbial perturbations precede or follow disease initiation and the contributory effect of gastrointestinal inflammation as shown previously [2, 4].

Studying the gut microenvironment in unaffected relatives of people with CD presents an opportunity to clarify the role of the gut microbiota in the aetiology of CD, in a population where genetics, environment and dietary risk exposures are likely to be similar to those of probands. Previous research in CD families has focused almost exclusively on the compositional differences of the faecal and mucosal microbiota but the findings remain inconsistent [5–10] and only a few studies provided a depiction of the global gut microbiota, using modern high-throughput sequencing techniques. As microbial composition does not reflect community function, there is now a pressing need to shift the paradigm from studies exploring compositional differences to those assessing functionality; the latter being potentially more relevant in health and disease. Studies which have explored both the composition and functionality of gut microbiota in IBD are currently lacking.

In this study, we hypothesised that the gut microbiota composition and functionality of unaffected relatives of patients with CD will be perturbed but the magnitude of observed alterations will be lower than those seen in their affected, genetically-linked kindred. For the first time in the literature we characterised the entire faecal bacterial community structure and genetic functional capacity, combining 16S rRNA gene amplicon and shotgun metagenomic sequencing, and measurement of major bacterial metabolites, repeatedly implicated in the pathogenesis of CD. We also attempted to reproduce the findings of previous similar research within the dataset produced in the current study.

## Material & methods

### Subjects & samples

Faecal samples were collected from 17 otherwise healthy, first-degree relatives of children with CD (CDR) and 14 controls unrelated to people with inflammatory bowel disease (HUC) (Table 1). A group of 19 children with CD on contemporary treatment, 16 of whom were blood-related to the CDR group, were used as a benchmark of the faecal ‘dysbiosis’ characteristics of CD (Table 1). All but two of the CDR participants were living in the same house with their CD relatives and one CD child was related to two participants from the CDR group. No participant had received antibiotics for at least two months prior to recruitment.

**Table 1:** Subject characteristics.

	CD (n = 19)	CDR (n = 17)	HUC (n = 14)
Gender, n: F/M	6/13	8/9	6/8
Age, y	12.2 (10.9:13.8)	40.0 (36.5:45.0)	39.6 (36.6:49.8)
FC, mg/kg	2055 (1083:2355)	66 (36:224)	<5 (5–28)
FC> 200 mg/kg, n (%)	17 (89)	4 (24)	0 (0)
CRP, mg/L	7 (7:13)		
ESR, mm/h	20.5 (10:39.3)		
Albumin, g/L	36.0 (32.5:38.5)		
PCDAI	15 (7.5:25)		
PCDAI < 10, n (%)	6 (35)		
*L2, n (%)	3 (16)		
*L2+L4, n (%)	5 (26)		
*L3, n (%)	2 (11)		
*L3+L4, n (%)	9 (47)		
Azathioprine, n (%)	11 (58)		
5-ASAs/methotrexate, n (%)	7 (37)		
Steroids, n (%)	4 (21)		
Biologics, n (%)	1 (5)		

Data are presented with median and interquartile ranges; CD: Children with Crohn’s disease, CDR: Unaffected blood relatives of children with Crohn’s disease, HUC: Healthy controls unrelated to patients with inflammatory bowel disease; FC: Faecal calprotectin; PCDAI: Paediatric Crohn’s Disease Activity Index

* Montreal classification.

### 16S rRNA gene amplicon sequencing

The composition of the gut microbiota was explored with amplicon sequencing of the 16S rRNA gene in the entire cohort. Bacterial DNA was isolated from faecal samples using bead-beating combined with the chaotropic method[11]. In brief, 200 mg of faeces were suspended in 250 μl of 4M guanidine thiocyanate (Sigma-Aldrich, UK), 0.1M Tris (pH 7.5) and 40 μl of 10% N-lauroyl sarcosine (Sigma-Aldrich, UK) buffer. After addition of 500 μl of 5% N-lauroyl sacrosine in 0.1 M phosphate buffer (pH 8) the sample was incubated at 70°C for 1 hour. Sterile zirconium beads (375 μl of 0.1 mm; Thistle Scientific, UK) were added and the bacterial cells were lysed (MP FastPrep 24, MP Biomedicals, California, USA) at 4.5 speed, three times for 60 sec. Polyvinylpolypyrrolidone (Sigma-Aldrich, UK) (15 mg) was added to the sample, which was vortexed and centrifuged at 15,000 × g at 4°C. The supernatant was collected and the pellet washed three times in total with 500 μl TENP (50 mM Tris [pH 8], 20 mM EDTA [pH 8], 100 mM NaCl, 1% polyvinylpolypyrrolidone (Sigma-Aldrich) and centrifuged for 3 min at 15,000 × g each time. Nucleic acids were precipitated by the addition of 1 ml of isopropanol for 10 min at room temperature and centrifuged for 5 min at 15,000 × g. The pellet was resuspended and pooled in 450 μl 100 mM phosphate buffer, pH 8, and 50 μl of 5 M potassium acetate (Sigma-Aldrich, UK). The sample was left in a fridge overnight. Following centrifugation at 15,000 × g for 30 min, the supernatant was collected and 2 μl of RNAse (RNAase ONE, Promega) added. The mixture was incubated at 37°C for 30 min. DNA was precipitated by the addition of 50 μl of 3 M sodium acetate (Sigma-Aldrich, UK) and 1 ml of ice-cold absolute ethanol. The sample was left for 30 min at room temperature or overnight at -20°C. DNA was recovered after centrifugation at 15,000 × g for 15 min, washed with 70% ice cold ethanol, dried, and resuspended in Tris-EDTA buffer. Sequencing of the V4 region of 16S rRNAgene was performed on the MiSeq (Illumina) platform using 2 × 250 bp paired-end reads as described previously[3].

### Shotgun metagenome sequencing

The genetic functional capacity of the gut microbiota was assessed in a random sample (using the RAND function of Microsoft Excel®) of 35 subjects (CD children, n = 11; CDR, n = 10; HUC, n = 14) with shotgun metagenomic sequencing of the entire bacterial genome on a HiSeq 2500 (Illumina) using the Nextera XT Prep Kit and Index kit and TruSeq Rapid SBS Kit reagents. Sequencing was performed following a paired-end 150 cycle recipe as described previously [3]. There was no significant difference in age or sex between the entire group and the subset used for metagenomic analysis [CDR: age p = 0.819, sex p = 0.729 and CD group: age p = 0.460, sex p = 0.387]

### Quantitative analysis of metabolites implicated in the pathogenesis of CD

The faecal short chain fatty acids (SCFA) acetate, propionate and butyrate, major anti-inflammatory and immunomodulatory bacterial metabolites [12], previously implicated in the aetiology of CD [13] were measured in diethyl ether extracts by gas chromatography using a TRACE™ 2000 gas chromatograph (ThermoQuest Ltd, Manchester, UK) equipped with a flame ionisation detector (250°C) and Zebron ZB-Wax capillary column (15 m x 0.53 mm x 1 μm), made of polyethylene glycol (Phenomenex, Cheshire, UK) [11]. The carrier gas was Nitrogen (30 ml/min). Internal standard (86.1 mmol/l, 3-methyl-n-valeric acid, Sigma-Aldrich, UK) and concentrated orthophosphoric acid were added to 50 mg of freeze-dried faecal material stored in 1M NaOH. The mixture was extracted three times with 3 ml diethyl ether, centrifuged and the ether layers pooled. One microlitre of ether extract was automatically injected (230°C, splitless) into the column. The column temperature was held at 80°C for 1 min, increased at 15°C/min until 210°C and held for 1 min. The chromatograms were analysed using Chrom-Card 32 version 1.07β5 (ThermoQuest, Milan, Italy). Authentic external standards were used as calibrators (166.5 mmol/l acetic, 135.0 mmol/l propionic, 113.5 mmol/l n-butyric, Sigma-Aldrich, UK). Results were presented per mass of faecal material (μmol/g) and as proportional ratio (%) to total SCFA.

### Bioinformatics analysis

#### 16S rRNA dataset

We trimmed and filtered the paired-end sequencing reads using Sickle v1.200[14] by applying a sliding window approach and trimming regions where the average base quality drops below 20. After this, we applied a 10bp length threshold to discard reads that fall below this length. We then used pandaseq (v 2.4) with a minimum overlap of 50bp to assemble the forward and reverse reads into a single sequence spanning the entire V4 region[15]. After having obtained the consensus sequences from each sample, we used the UPARSE (v7.0.1001) as described in https://bitbucket.org/umerijaz/amplimock/src for operational taxonomic unit (OTU) construction. The approach was as follows: we pooled the reads from different samples together and added barcodes to keep an account of the samples these reads originated from. We then de-replicated the reads and sorted them by decreasing abundance and discarded singletons. Reads were then clustered based on 97% similarity, discarding reads shorter than 32bp. Even though the cluster_otu command in usearch removes reads that have chimeric models built from more abundant reads, a few chimeras may be missed, especially if they have parents that are absent from the reads or are present in very low abundance. Therefore, in the next step, we used a reference-based chimera filtering step using a gold database (http://drive5.com/uchime/uchime_download.html) that is derived from the ChimeraSlayer reference database in the Broad Microbiome Utilities (http://microbiomeutil.sourceforge.net/). The original barcoded reads were matched against clean OTUs with 97% similarity (a proxy for species level separation) to generate OTU tables for different samples. The representative OTUs were then taxonomically classified against the RDP database using the standalone RDPclassifier v2.6[16] with the default—minWords option of 5. To find the phylogenetic distances between OTUs, we first multisequence aligned the OTUs against each other using mafft v7.040[17] and then used FastTree v2.1.7 on these alignments to generate an approximately-maximum-likelihood phylogenetic tree[18].

#### Shotgun metagenomics sequencing dataset

Whole-genome shotgun metagenomics reads were trimmed for Nextera adaptors and low-quality ends using Trimmomatic[19]. These were screened against the hg18 human reference genome using Bowtie2[20], with any matching sequences discarded. Reads were subsampled to 2 million reads and assigned to functional categories through alignment to Kyoto Encyclopedia of Genes and Genomes (KEGG) release 58.0 (April 1, 2011) using RAPSearch2 using a translated nucleotide to amino acid search. Alignments were assigned to KEGG metabolic pathways using HUMAnN[21]. HUMAnN uses KEGG orthology as well as orthologus families of genes and calculates coverages as pathway presence/absences. It also uses MinPath[22] to filter out pathways that have very little evidence.

### Statistical analysis

Statistical analysis was performed in R software and was similar for the 16S rRNA and metagenomic datasets unless otherwise stated. Where appropriate, the abundance data was normalised[23] choosing log-relative transformation before doing statistics for downstream analysis. To find OTUs that are significantly different between the conditions, we used the DESeq2 package[24]. This uses a negative binomial to model the abundance data (OTU frequencies) and empirical Bayes to shrink OTU-wise dispersions to identify OTUs that have the maximum log-fold changes between different conditions. Differential expressions were tested by performing a Wald test on shrunken log-fold changes adjusted for multiple comparisons. For community analysis (including alpha and beta diversity analyses) we used the Vegan package[25], in particular the two functions *adonis* for PERMANOVA and *betadisper* for the analysis of multivariate homogeneity of group dispersions. The p-values reported in such a case were those returned by the functions themselves. Microbial compositional structure was assessed using non-metric multidimensional scaling plot (NMDS). We applied the Bray-Curtis dissimilarity index, which considers bacterial taxon presence and abundance, but also the unweighted Unifrac distance analysis which takes into account the phylogenetic distances (relatedness) of the bacterial taxa, without accounting for their abundance. Specifically, the abundance table was converted to a presence/absence table in the case of unweighted Unifrac distance. The taxa present in one or both samples were then placed on the phylogenetic tree. The distance between two samples was then calculated as the sum of unshared (taxa not common) branch lengths divided by the sum of all tree branch lengths, both shared (taxa common) and unshared, between pair of samples. To calculate Unifrac distances, we used the Phyloseq[26] package. We also performed local contribution for β-diversity (LCBD) analysis to measure the contribution of each sample to the total OTU β-diversity, calculated from all study samples together (% of total community dispersion) [27]. Samples with high LCBD represent samples that are markedly different from the average β-diversity of all study samples. For differences in metagenomic metabolic pathways, we used the Kruskal-Wallis test. For correlations between SCFA and discriminatory OTUs, we used Kendall rank correlation. For α-diversity, subanalysis was also performed accounting for the genetic relatedness of participants from the CD and CDR group (paired data). We used the Benjamini-Hochberg correction for multiple testing in all analyses. The authors maintain the general scripts as well as tutorials for the above analyses at http://userweb.eng.gla.ac.uk/umer.ijaz#bioinformatics.

### Ethical considerations

Approval to conduct the study was granted by the Yorkhill Hospital Research Ethics Committee (Reference Number: 05/S0708/66). Carers and patients provided written informed consent.

## Results

### Faecal calprotectin

Faecal calprotectin (mg/kg) was significantly higher in CD children (median; IQR: 2,055; 1,083–2,355; p<0.001) and CDR (median; IQR: 66; 36–224; p<0.001) than HUC (median; IQR: <5; <5–28). Four out of the 17 (23%) unaffected relatives had calprotectin values above 200 mg/kg. Faecal calprotectin was within the reported normal adult range (<50 mg/kg) in all samples from the HUC group (Table 1). Six children with CD (35%) had a Paediatric Crohn’s Disease Activity Index less than 10 suggestive of clinical disease remission (Table 1).

### Faecal microbiota community structure

**Fig 1:**
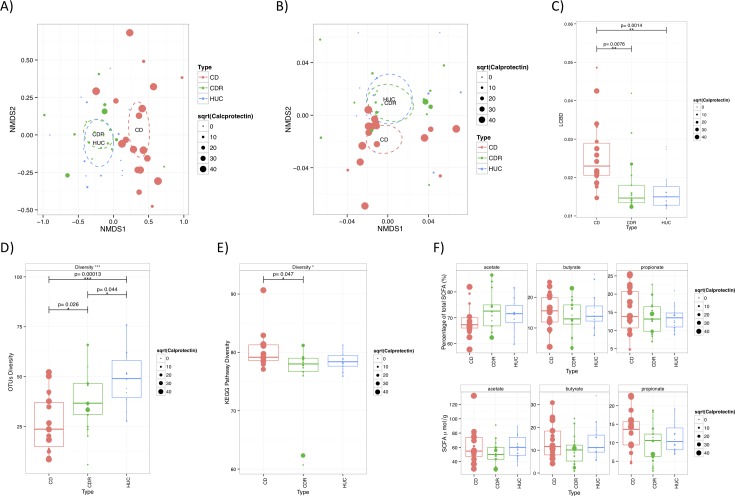
Microbiota composition and functionality characteristics of unaffected relatives of children with Crohn’s disease, their unaffected relatives and healthy controls with no familial history of IBD. A) Non-metric multidimensional scaling (NMDS) plot using Bray-Curtis dissimilarity index which considers bacterial taxon presence and abundance. B) Non-metric multidimensional scaling (NMDS) plot using Unifrac phylogenetic distances which takes into account the phylogenetic distances (relatedness) of the bacterial taxa, without accounting for their abundance; The ellipses in A and B represent the 95% confidence intervals based on the standard errors of the average of the axis scores for each group using the ordiellipse function of the R's vegan package C) Local contribution of β-diversity (LCBD) analysis which considers the contribution of each sample to the total OTU β-diversity, calculated from all study samples together (% of total community dispersion); D) Shannon α-diversity (expressed in richness equivalents) based on operational taxonomic unit assignments (OTU) (p = 0.039 when accounting for the genetic relatedness of the participants in the CDR and CD group using paired data analysis); E) Diversity of KEGG metabolic pathways based on metagenomics sequencing (p = 0.127 when accounting for the genetic relatedness of the participants in the CDR and CD group using paired data analysis); F) Faecal short chain fatty acids (μmol/g and % of total SCFA); The size of the dot is proportional to the concentration of faecal calprotectin; CD: Children with Crohn’s disease, CDR: Unaffected blood relatives of children with Crohn’s disease, HUC: Healthy controls unrelated to patients with inflammatory bowel disease.

When we looked at microbiota community structure (β-diversity), there was no difference between the CDR and HUC groups, but both of these were distinct from the communities of CD. This was evident from the NMDS plot for the Bray-Curtis dissimilarity index (Fig 1, Panel A) and the unweighted Unifrac distance analysis (Fig 1, Panel B) which takes into account the phylogenetic relatedness of the bacterial taxa, without accounting for their abundance. LCBD analysis showed that the gut microbiota structure of CD individuals was different to the microbiota of the other two groups, but no difference was seen between the CDR and HUC (Fig 1 Panel C).

Using the *betadisper* function of the R vegan package, the variation in community structure among samples of the same participant group (measured as the distance of each sample from their respective group ellipse centroid), was significantly different between the three groups for bacterial taxon abundance (i.e. Bray-Curtis) but not their phylogenetic relatedness (unweighted Unifrac). This significant difference in Bray-Curtis similarity index was due to a higher variation in the microbial community structure of the samples of the CD group compared with the samples from the two adult groups where community structure was more homogeneous (Bray-Curtis, CD vs CDR, p = 0.035; CD vs HUC, p<0.001). Notably, within the CDR group, where faecal calprotectin varied among subjects, there was a tendency for the community structure of the gut microbiota of subjects with increased faecal calprotectin to diverge from those with normal levels (Bray-Curtis, p = 0.07; unweighted Unifrac, p = 0.14) (Fig 1, Panel A & B).

Both groups (CDR and HUC) presented a significantly higher (both p<0.05) microbiota diversity richness and evenness (Shannon α-diversity index) than CD (Fig 1, Panel D). Between them, the microbiota diversity was lower in the CDR compared with the HUC groups (p = 0.044) (Fig 1, Panel D). Results remained the same when analysis was performed considering the genetic relatedness of the participants in CDR and CD groups (p = 0.039). There was no significant correlations between the α-diversity index with the concentration of faecal calprotectin in all three groups (CD, p = 0.440; CDR, p = 0.901; HUC, p = 0.440).

### Bacterial taxon relative abundance

The relative abundance of 89 OTUs were significantly different between the CD and CDR group (S1 Fig). Collectively, and in close agreement with previous research, which compared CD microbiota characteristics with unrelated controls [3, 28], we observed a reduction in the abundance of OTUs belonging predominantly to *Ruminococcaceae*, *Lachnospiraceae*, as well as in the genera *Phascolarctobacterium*, *Parabacteroides*, *Akkermansia* and *Methanobrevibacter*. Likewise a respective increase was observed in OTUs from *Enterobacteriaceae*, *Pasteurellaceae*, *Veillonella*, *Dorea*, *Anaerostipes*, *Enterococcus* genera, *Clostridium_XVIII* and *Clostridium_XlVa* clusters, in CD compared with their genetically linked kindred (S1 Fig).

**Fig 2:**
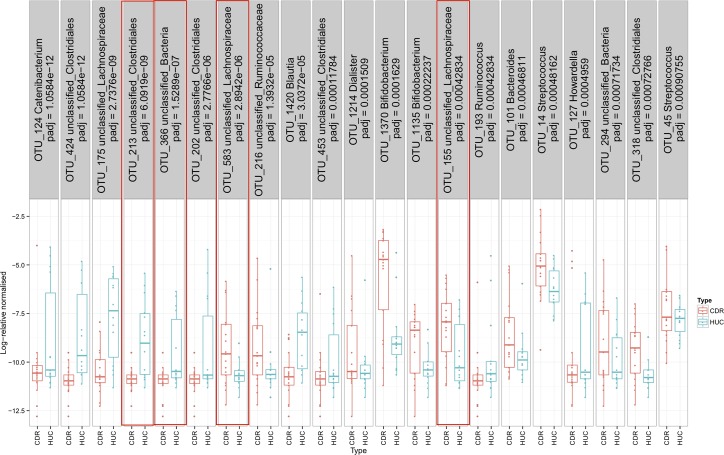
Log-relative abundances of OTUs which differentiated unaffected relatives of children with Crohn’s disease and healthy controls with no familial history of the disease. Taxonomic classification is given at the highest level of phylogenetic resolution. Within red box frames those OTUs which were identical to the ones which differed between children with CD and healthy paediatric controls in a previous study [3]^.^ CDR: Unaffected blood relatives of children with Crohn’s disease, HUC: Healthy controls unrelated to patients with inflammatory bowel disease.

At individual bacterial taxon level, the relative abundance of 21 of 1,243 OTUs (1.8% of all) was significantly different between the CDR and HUC groups (Fig 2). This proportion was lower than the number of OTUs which differed between CD and CDR (89 of 1,159; 7.7%) or HUC group (113 of 1,180; 9.6%). From the 21 OTUs which differentiated CDR from HUC, none was significantly associated with calprotectin within the former group. The relative abundance of OTUs which belonged to the *Catenibacterium* spp., *Clostridiales*, *Lachnospiraceae*, *Blautia* genus, *Ruminococcus* genus and *Howardella* spp. were significantly lower in the CDR than the HUC group (Fig 2). In contrast, OTUs belonging to *Clostridiales*, *Lachnospiraceae*, *Ruminococcaceae*, *Dialister*, *Bifidobacterium* and *Streptococcus* genera were significantly more abundant in CDR participants (Fig 2).

When we compared these 21 discriminatory OTUs with those we described previously in the literature [3], between children with CD and their age matched controls, four of these OTUs were identical (Fig 2). With reference to their nearest taxonomic classification, all these OTU belonged to the *Clostridiales* order. Two of them were significantly higher and two significantly lower in the CDR compared with the HUC group (Fig 2). One of them, *Coprococcus eutactus*, a butyrate producer which was significantly higher in the unaffected relatives, was resolved at 100% identity and 100% coverage in the NCBI database. None of these OTUs was significantly associated with calprotectin within the CDR group.

### Cross-validation of results from previous research

When we attempted to reproduce findings of previous similar research by other groups within our own dataset[7, 8], no consensus was found for most candidate taxa tested, except for species belonging to *Collinsella*. Three of the eight OTUs assigned to this taxon were significantly lower in CDR than HUC participants, a finding which accords to observations made by Joossens et al for *Collinsella aerofaceins*[8]. In contrast to the results by Hedin et al[7], no significant differences were observed between CDR and HUC for nine OTUs assigned to *Roseburia* and six OTUs assigned to *Faecalibacterium prausnitzii*. From the 11 OTUs which were assigned to *Ruminococcus*, one differed between CDR and HUC but the direction of this change was opposite to the one reported previously for *Ruminococcus torques* [8].

### Genetic functional capacity and bacterial metabolites

When metagenomic reads were assigned to KEGG metabolic pathways with HUMAnN, no significant difference in gene richness equivalents, were observed between CDR and HUC (Fig 1, Panel E). None of the metabolic pathways differed between these two groups after adjusting for multiple testing. In contrast, the genetic functional capacity of the CDR and HUC groups was (CD vs CDR, p = 0.047) or tended to be higher (CD vs HUC, p = 0.081) than that of CD and in accordance with our previous research (Fig 1, Panel E) [3]. In pairwise comparison between groups, the relative abundance of two KEGG pathways involved in aminoacyl-tRNA biosynthesis and ribosome function were significantly lower in CD than CDR patients (S2 Fig).

With regard to SCFA, no significant differences were found in the concentration or proportional ratio (%) of any of the three major SCFA between the three groups (Fig 1, Panel F). Among the discriminatory OTUs, between CDR with CD, the percentage propionate in CD participants was negatively correlated with two OTUs belonging to *Veillonella* and positively with an OTU from *Phascolarctobacterium* (S3 Fig), a genus which includes species which convert succinate to propionate [29]. No significant correlations were observed between any of the SCFA with OTUs which discriminated the CDR from the HUC group, after correcting for multiple testing (S4 Fig).

## Discussion

In this study, we observed fewer, than anticipated, perturbations in the composition and functionality of the microbiota of unaffected relatives of patients with CD. Healthy status was the strongest microbiota composition classifier. The CDR microbiota was significantly different to that of their relatives with CD and the taxon alterations we observed were similar to those reported between CD patients and unrelated controls in independent literature [28]. By inference, these findings suggest that an extensively altered microbiota, characteristic of CD dysbiosis, is not evident in genetically-linked unaffected relatives. This finding accords with previous research where the faecal microbial communities were found to be more similar between healthy identical twins than between twins with CD, especially those who were discordant for the disease[5]. Importantly, the observed perturbations in the microbiota of CDR participants were associated with composition rather than genetic functional capacity or the pattern of SCFA. These findings point towards a preservation of the microbial community functional capacity despite some alterations in microbiota composition in unaffected relatives of CD patients.

While we have identified 21 taxa which differentiated the CDR from the HUC participants, this was a small fraction of the overall community and the extensive differences observed when each of these groups was compared with CD. Furthermore, as these taxa were not associated with faecal calprotectin, their role in the aetiology of CD may be less important. Replication of these findings by future research is required to infer their causal association to CD pathogenesis.

Comparison of the results of this study with previous research is not trivial but rather challenging, considering the differences among studies in the methodology used (particularly with regards to DNA extraction, 16S region amplified, sequencing methodology and bioinformatic approach) and the subsequent depth of taxonomic resolution that each study applied to characterise the bacterial composition. In this study, we characterised bacterial taxa approximating species level and our results suggest that discriminant OTUs belonging to the same phylogenetic order or family were not only different between the CDR and HUC groups but the direction of this difference was not always the same

Alterations in specific taxa presented in previous research, were replicated only for species belonging to *Collinsella* [5–7]. However, the less diverse microbiota we observed in the CDR participants is in accordance with the results of Hedin, *et al* in siblings of patients with CD [6].

A tendency for the community structure of CDR participants with raised faecal calprotectin to diverge further than those of their counterparts with no colonic inflammation is intriguing and agrees with previous research[6]. This finding may indicate a transient effect of colonic inflammation on gut microbiota composition[2, 4] or the reverse, activation of the immune system by an unstable and perturbed microbiota. Whether or not it suggests a mid-way point from health to the development of Crohn’s disease warrants further exploration. Biomarkers of disease susceptibility in health relatives would be a helpful clinical tool to allow the development of preventative strategies. In this study we did not explore whether raised levels of calprotectin and an associated perturbed microbiota persisted over time, nor the proportion of relatives who developed CD later in life. Ongoing large scale multicentre studies http://www.gemproject.ca/, prospectively following subjects at high risk of CD development will be well placed to unravel this important observation and its clinical relevance.

Collectively, these early data suggest that the distinct microbiota ‘dysbiosis’, consistently observed in people with CD, occurs to a much lower extent in their healthy genetically-linked counterparts. It is therefore possible that major loss of ‘normobiosis’ either occurs close to disease onset or is a secondary effect of the illness [10].

The main limitation of this study is the modest number of participants, albeit comparable to or larger than previous reports [5–7, 10]. The inherent cost and complexity of multi-omics research at present tends to limit sample size for exploratory research such as this study. We have nonetheless corrected for multiple testing in our analysis to avoid type I error, however in doing so we may have introduced a type II error in some of our statistical analyses. CD patients were not controlled for medication use, disease phenotype or disease activity. Each of these might have affected their microbiota [30] and confounded the results of this study. Inclusion of an adult cohort of CD patients, blood-related to unaffected relatives, would have been ideal in this study but unfortunately this was not available and an age difference may have confounded some of our results. None of the participants were on antibiotics or exclusive enteral nutrition, however, both of which have shown clear effects on microbiota in previous research [3].

In conclusion, some alterations were observed in the gut microbiota of unaffected relatives of CD children but a distinct microbial ‘dysbiosis’ similar to that observed in CD was not evident. Further consideration should be given to describing the microbial change seen during transition from health to establishment of CD.

## Supporting information

S1 Fig Log-relative abundances of OTUs which differentiated children with CD from their unaffected relatives. 1: Taxonomic classification is given at the highest level of phylogenetic resolution. CDR: Unaffected blood relatives of children with Crohn’s disease; CD: children with CD
(PDF)

S2 Fig Log-relative abundances of KEGG pathways which differentiated children with CD from their unaffected relatives. CDR: Unaffected blood relatives of children with Crohn’s disease; CD: children with CD; KEGG: Kyoto Encyclopedia of Genes and Genomes
(PDF)

S3 Fig Kendal rank correlations between individual SCFA and the relative abundance of discriminatory OTUs between CD and CDR. CDR: Unaffected blood relatives of children with Crohn’s disease; CD: children with CD; SCFA: Short Chain Fatty Acids
(PDF)

S4 Fig Kendal rank correlations between individual SCFA and the relative abundance of discriminatory OTUs between CDR and HUC. CDR: Unaffected blood relatives of children with Crohn’s disease; HUC: Healthy controls unrelated to patients with inflammatory bowel disease. SCFA: Short Chain Fatty Acids
(PDF)

S1 Table Metadata associated with the samples and description of sequencing data. (CSV)
