# Supplementary figures and images for: The distinct features of microbial ‘dysbiosis’ of Crohn’s disease do not occur to the same extent in their unaffected, genetically-linked kindred
Source: PLoS One. 2017 Feb 21;12(2):e0172605. doi: 10.1371/journal.pone.0172605 (PMC5319678; doi:10.1371/journal.pone.0172605)

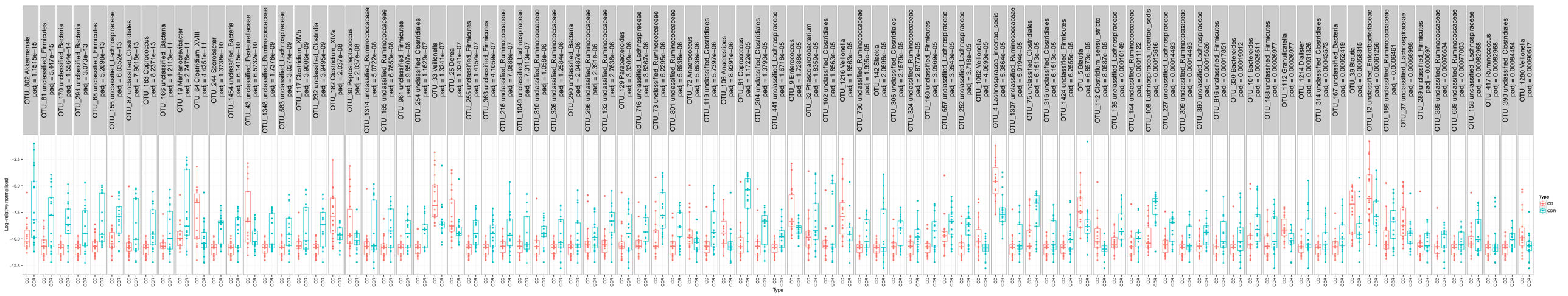

Supplement: S1 Fig — 1: Taxonomic classification is given at the highest level of phylogenetic resolution. CDR: Unaffected blood relatives of children with Crohn’s disease; CD: children with CD (PDF) [file pone.0172605.s001.pdf]

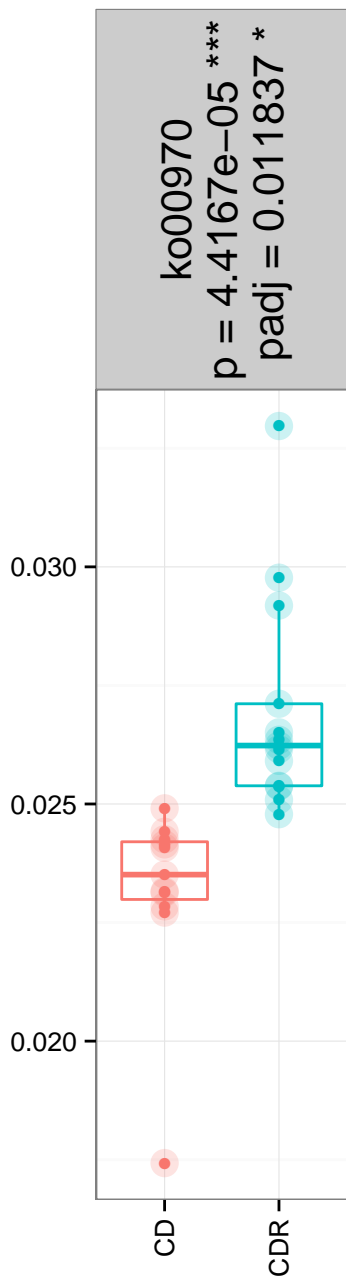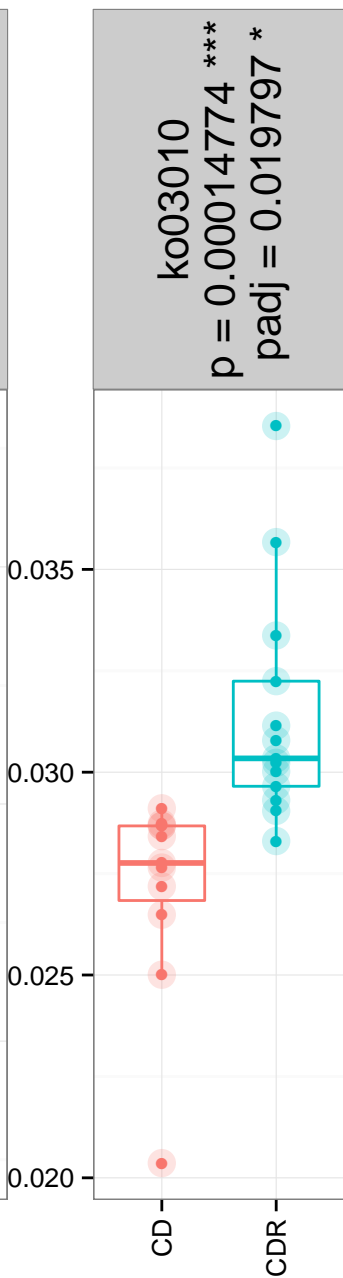

Type

CD

CDR

Supplement: S2 Fig — CDR: Unaffected blood relatives of children with Crohn’s disease; CD: children with CD; KEGG: Kyoto Encyclopedia of Genes and Genomes (PDF) [file pone.0172605.s002.pdf]

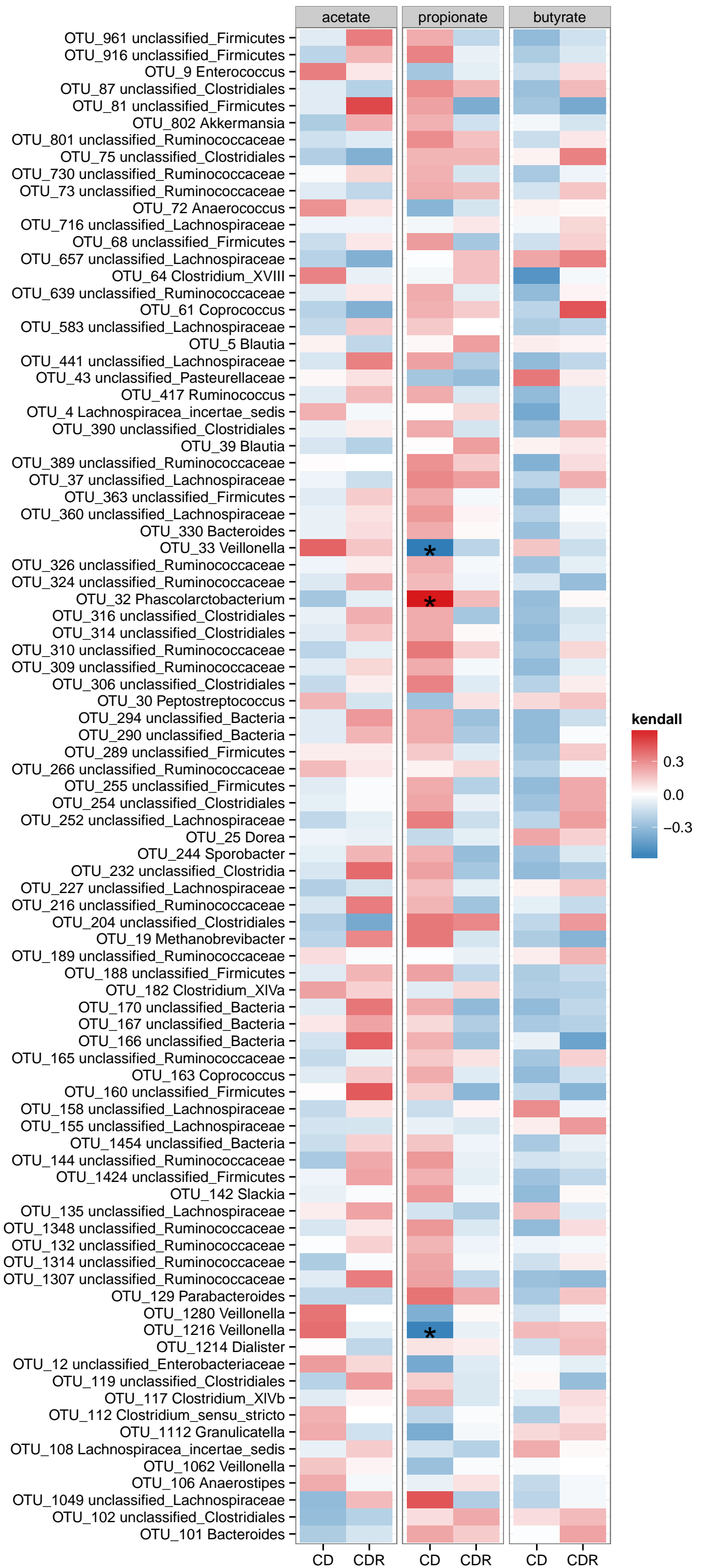

Supplement: S3 Fig — CDR: Unaffected blood relatives of children with Crohn’s disease; CD: children with CD; SCFA: Short Chain Fatty Acids (PDF) [file pone.0172605.s003.pdf]

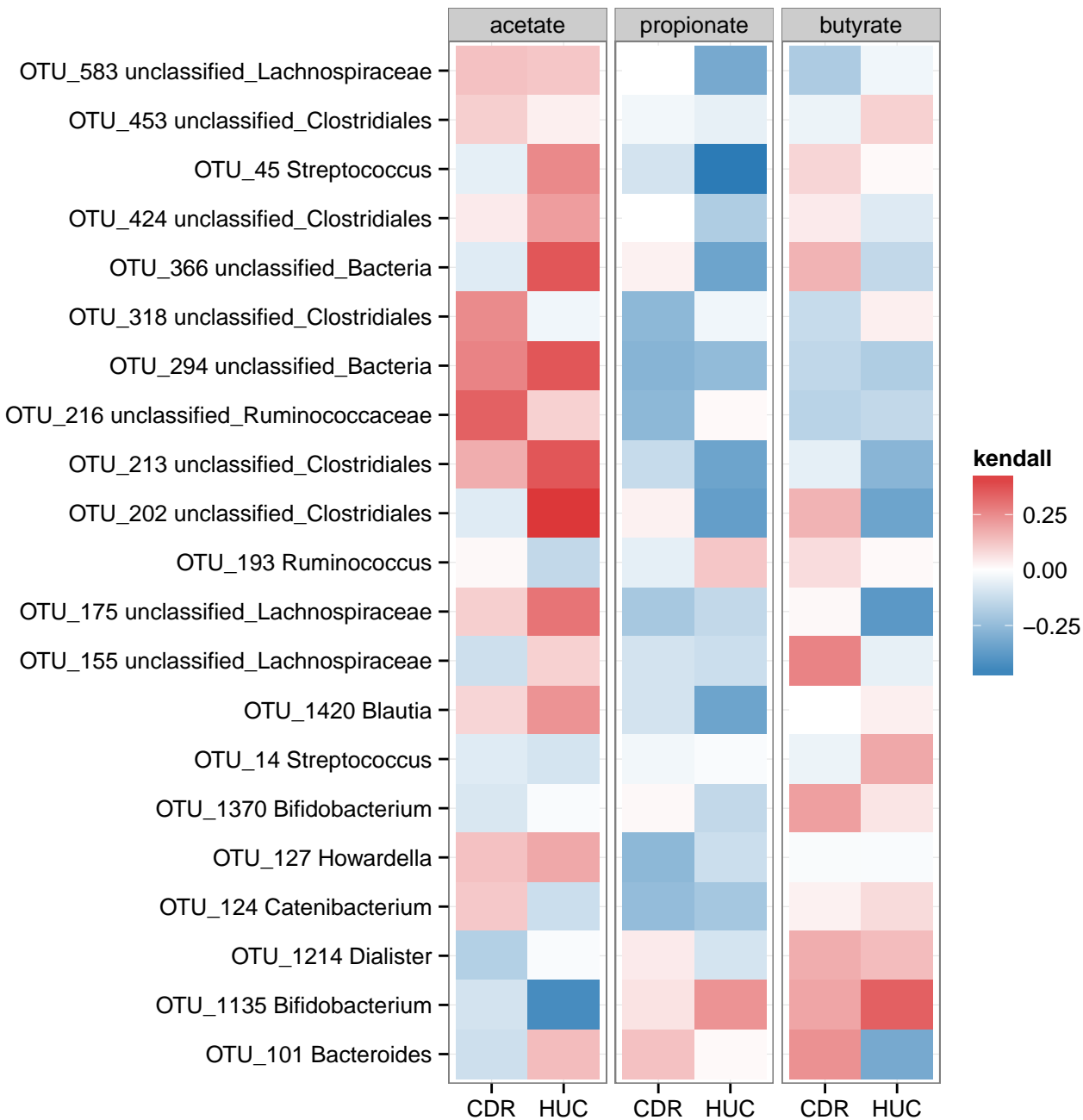

Supplement: S4 Fig — CDR: Unaffected blood relatives of children with Crohn’s disease; HUC: Healthy controls unrelated to patients with inflammatory bowel disease. SCFA: Short Chain Fatty Acids (PDF) [file pone.0172605.s004.pdf]
